# A shotgun metagenomic analysis of the fecal microbiome in humans infected with *Giardia duodenalis*

**DOI:** 10.1186/s13071-023-05821-1

**Published:** 2023-07-18

**Authors:** Brett A. McGregor, Elham Razmjou, Hossein Hooshyar, Drew R. Seeger, Svetlana A. Golovko, Mikhail Y. Golovko, Steven M. Singer, Junguk Hur, Shahram Solaymani-Mohammadi

**Affiliations:** 1 Department of Biomedical Sciences, School of Medicine and Health Sciences, University of North Dakota, Grand Forks, ND USA; 2 Department of Parasitology and Mycology, School of Medicine, Iran University of Medical Sciences, Tehran, Iran; 3 Department of Medical Parasitology and Mycology, School of Medicine, Kashan University of Medical Sciences, Kashan, Iran; 4 Department of Biology, Georgetown University, Washington, DC USA; 5 Laboratory of Mucosal Immunology, Department of Biomedical Sciences, School of Medicine and Health Sciences, University of North Dakota, Grand Forks, ND USA

**Keywords:** Human, Giardiasis, Gut, Microbiome, Infection, Mucosal

## Abstract

**Background:**

The mechanisms underlying the clinical outcome disparity during human infection with *Giardia duodenalis* are still unclear. In recent years, evidence has pointed to the roles of host factors as well as parasite’s genetic heterogeneity as major contributing factors in the development of symptomatic human giardiasis. However, it remains contested as to how only a small fraction of individuals infected with *G. duodenalis* develop clinical gastrointestinal manifestations, whereas the majority of infected individuals remain asymptomatic. Here, we demonstrate that diversity in the fecal microbiome correlates with the clinical outcome of human giardiasis.

**Methods:**

The genetic heterogeneity of *G. duodenalis* clinical isolates from human subjects with asymptomatic and symptomatic giardiasis was determined using a multilocus analysis approach. We also assessed the genetic proximity of *G. duodenalis* isolates by constructing phylogenetic trees using the maximum likelihood. Total genomic DNA (gDNA) from fecal specimens was utilized to construct DNA libraries, followed by performing paired-end sequencing using the HiSeq X platform. The Kraken2-generated, filtered FASTQ files were assigned to microbial metabolic pathways and functions using HUMAnN 3.04 and the UniRef90 diamond annotated full reference database (version 201901b). Results from HUMAnN for each sample were evaluated for differences among the biological groups using the Kruskal–Wallis non-parametric test with a post hoc Dunn test.

**Results:**

We found that a total of 8/11 (72.73%) human subjects were infected with assemblage A (sub-assemblage AII) of *G. duodenalis*, whereas 3/11 (27.27%) human subjects in the current study were infected with assemblage B of the parasite. We also found that the parasite’s genetic diversity was not associated with the clinical outcome of the infection. Further phylogenetic analysis based on the *tpi* and *gdh* loci indicated that those clinical isolates belonging to assemblage A of *G. duodenalis* subjects clustered compactly together in a monophyletic clade despite being isolated from human subjects with asymptomatic and symptomatic human giardiasis. Using a metagenomic shotgun sequencing approach, we observed that infected individuals with asymptomatic and symptomatic giardiasis represented distinctive microbial diversity profiles, and that both were distinguishable from the profiles of healthy volunteers.

**Conclusions:**

These findings identify a potential association between host microbiome disparity with the development of clinical disease during human giardiasis, and may provide insights into the mechanisms by which the parasite induces pathological changes in the gut. These observations may also lead to the development of novel selective therapeutic targets for preventing human enteric microbial infections.

**Graphical Abstract:**

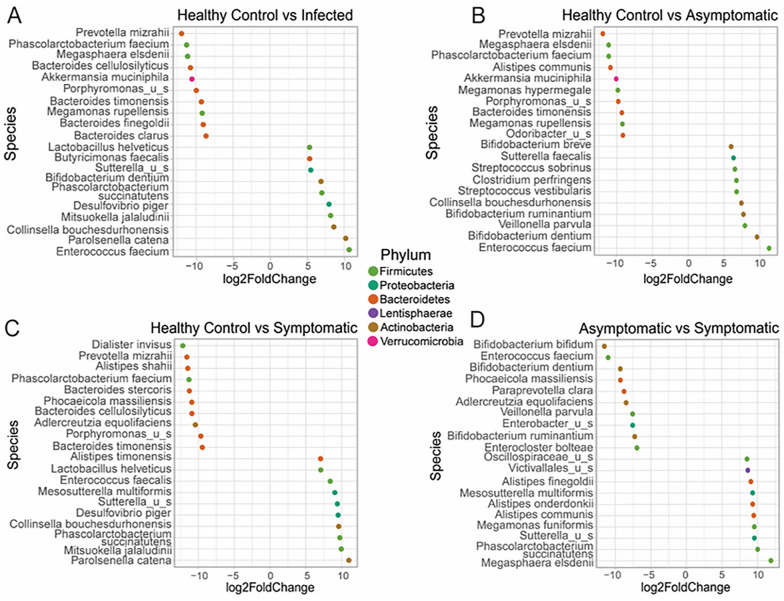

**Supplementary Information:**

The online version contains supplementary material available at 10.1186/s13071-023-05821-1.

## Background

Human enteric microbial infections are considered a leading cause of morbidity and mortality in children under the age of 5 in underdeveloped and developing nations, leading to 1.3 million deaths per year [1–4]. Human giardiasis, caused by *Giardia duodenalis*, is one of the most commonly identified intestinal protozoan infections, with 280 million new cases annually worldwide [5]. The prevalence rates of human giardiasis vary greatly from 20 to 30% in developing countries to up to 5% in developed nations [6, 7]. Human giardiasis is the most commonly identified intestinal parasitic disease in the United States, with one million estimated human giardiasis cases reported annually [8–10]. Infection with *G. duodenalis* is associated with a wide range of clinical outcomes. Most cases of human giardiasis are associated with no overt clinical symptoms, whereas others report severe gastrointestinal symptoms, accompanied by a nutrient malabsorption syndrome [2, 3, 11–13]. Despite the widespread distribution of human giardiasis, very little is known about the mechanisms responsible for these variable outcomes.

*Giardia duodenalis* is a genetically heterogeneous group that has been divided into eight clades (A–H), referred to as assemblages, based on sequences of the 18S ribosomal RNA (rRNA) and housekeeping loci such as triose phosphate isomerase (*tpi*), glutamate dehydrogenase (*gdh*), and β-giardin (*bg*) [14]. Only assemblages A and B are commonly observed in humans, although both are also identified in many other mammalian hosts [15–17]. Multiple studies have investigated a potential association between parasite genotype/assemblage and the clinical or immunopathological outcomes of human giardiasis [18–21]. Some studies have documented a direct correlation between a given genotype/assemblage and the induction of immunopathological alterations in the gut following human and murine giardiasis [22–24], whereas others have failed to establish such an association [25–28]. The lack of a direct association between those loci used for the assemblage identification and the propensity of a given genotype/assemblage to induce gut immunopathology could potentially explain the disparity in the outcomes of murine and human giardiasis [2].

Over the last few decades, several hypotheses have been suggested to account for the variations observed in the clinical outcomes of *G. duodenalis* infections in humans [reviewed in (2,3)]. The ability of a given *G. duodenalis* isolate to elicit T cell-dependent immune responses has been proposed as a mechanism by which immunopathological changes, including disaccharide deficiency, are induced [2, 3, 29–31]. Mice deficient in CD8^+^ T cells (beta-2 microglobulin [*β2m*]^*−*/*−*^) failed to develop disaccharidase deficiency (e.g., sucrase deficiency) following *G. duodenalis* infection, whereas the ability of these mice to clear the parasite was unaffected [11]. Consistent with these findings, the adoptive transfer of mucosal CD8^+^ T cells, but not CD4^+^ T cells, isolated from *Giardia muris*-infected donors was sufficient to induce mucosal injuries, including loss of microvilli surface area and reduced disaccharidase activity, in T cell-deficient recipients as compared with their controls [32, 33]. Further investigations demonstrated that the activation of pathogenic CD8^+^ T cells was dependent on the microbiome and that the treatment of *G. duodenalis*-infected mice with an antibiotic cocktail abolished the induction of disaccharidase deficiency as compared with controls [34]. These observations suggest a critical role played by the host microbiome in the regulation of immunopathological sequelae in the gut during giardiasis.

Isogenic mice obtained from different commercial vendors had varied susceptibility to *G. duodenalis* infection, and the resistance to infection was readily transferable to the susceptible mice upon being co-housed before *G. duodenalis* infection [35]. Notably, the transfer of the fecal microbiome from human subjects with symptomatic giardiasis into conventional mice led to increased pathogenicity of *G. duodenalis* and more severe immunopathological alterations in these mice as compared with germ-free animals [36]. Further studies have established an intimate crosstalk between the mammalian host, the microbiome, and the outcome of *Giardia* spp. infection [37–40]. These observations suggest critical roles played by the microbiome in determining host susceptibility and parasite pathogenicity during giardiasis.

The microbiome undergoes drastic alterations following infections with both assemblages A and B of *G. duodenalis*, and these changes depend on the host’s genetic background [41, 42]. These alterations in the microbiome are not restricted to the proximal portions of the small intestine, where *G. duodenalis* mainly dwells, but are observed throughout the entire intestinal tract of infected hosts and can persist even after parasite clearance [43, 44].

Prebiotic or probiotic supplementation prior to or concurrent with *G. duodenalis* infection resulted in significant attenuation in the severity of giardiasis, modulation of immune responses, and restored morphological abnormalities associated with *G. duodenalis* infection in mice [45, 46]. Animals receiving a high-fiber diet had enhanced secretion of the intestinal mucus and were more resistant to *G. duodenalis* infection when compared with control mice fed a low-fiber diet [47]. Those maintained on a diet with high fat content, however, were more susceptible to *G. duodenalis* infection with signs of mucus layer disruption, goblet cell hyperplasia, and microbiome dysbiosis [48]. These observations demonstrate that the exogenous dietary prebiotic is likely to contribute to the pathogenesis of giardiasis by regulating the parasite’s virulence factors and/or modulating host immune responses.

Here we investigated the association between the parasite’s genetic diversity/microbiome diversity and the development of clinical symptoms in human subjects with *G. duodenalis* infections. Our findings reveal a previously lesser-known role played by the host microbiome and its association with clinical symptoms during human giardiasis. The understanding of the mechanisms by which *G. duodenalis* interacts with the host microbiome, as well as the crosstalk between host and parasite factors, will provide insights into novel mechanisms by which the parasite induces pathological changes and may further demonstrate the mechanisms underlying variations in the clinical outcome observed during human giardiasis. Defining the crosstalk between the pathogen and host factors may further provide protective and therapeutic targets for the control of human *G. duodenalis* infections.

## Methods

### Study subjects

Fecal specimens were collected from healthy volunteers and individuals infected with *G. duodenalis* in an area endemic for human giardiasis in central Iran. The demographics of participants in this study are presented in Table 1. The demographics (mean ± SEM) were analyzed using GraphPad Prism software (version 8.4.0; GraphPad, San Diego, CA).

**Table 1:** Study group demographics

	Age (years)			Sex	
	Median	Mean ± SEM	Range (years)	Female (%)	Male (%)
Human subjects (*n* = 17)	32	33.53 ± 5.25	5–73	4 (23.52)	13 (76.48)

*mean* ± *SEM*, mean ± standard error of the mean

Infected individuals with asymptomatic giardiasis with no prior signs of symptomatic disease were included, whereas individuals with symptomatic giardiasis mainly experienced gastrointestinal symptoms. Infected human subjects were initially defined as individuals positive for *G. duodenalis* trophozoites/cysts using stool microscopy on saline wet mount fecal preparations, confirmed by a subsequent positive stool-based polymerase chain reaction (PCR) assay using sets of species-specific primers for *G. duodenalis*. The healthy controls were enrolled from individuals being negative for *G. duodenalis* using stool microscopy on saline wet mount fecal preparations, followed by a negative stool-based PCR assay for *G. duodenalis*. No other pathogenic protozoan or helminth parasites were detected in the feces of these individuals.

### Ethics statement

Written informed consent was obtained from all participants or their parents/legal representatives in the case of minors. This study was approved by the Ethical Committee of the Kashan University of Medical Sciences in accordance with the Iranian Ministry of Health, Treatment and Medical Training Protection Code of Human Subjects in Medical Research. The institutional biosafety committees of the University of North Dakota School of Medicine and Health Sciences (#IBC-202103-026) and Georgetown University (#IBC-27-18) reviewed and approved this study.

### DNA preparation and stool-based multilocus sequence typing (MLST) of *G. duodenalis* isolates using next-generation sequencing (NGS) of the *tpi*, *gdh*, and *bg* loci

Total genomic DNA (gDNA) extraction from fecal samples for MLST of *G. duodenalis* isolates was performed by Molecular Research LP (Shallowater, TX, USA) as described previously [49]. The oligonucleotide primers AL3543 (external forward), AL3546 (external reverse), AL3544 (internal forward), and AL3545 (internal reverse) were used to specifically PCR-amplify a 530-base-pair (bp) fragment within the *tpi* locus of *G. duodenalis* as described earlier (Additional file 1: Table S1) [49, 50]. PCR amplifications were performed in 50 µl reaction volumes as described [49]. The PCR amplification program consisted of an initial template denaturation step of 5 min at 95 °C, followed by 35 amplification cycles of 45 s at 94 °C, 45 s at 50 °C, and 60 s at 72 °C with a final extension of 10 min at 72 °C. In the second PCR reaction, the annealing temperature was increased to 58 °C, while other parameters were left unaltered [51, 52].

*Giardia duodenalis* mixed infections (assemblages A+B) were identified by targeting the *tpi* locus of *G. duodenalis* using a nested-PCR strategy as described earlier [51, 53]. The primary PCR amplification program was identical to that described above, whereas the second PCR reaction was performed using assemblage-specific oligonucleotide primers Af and Ar (assemblage A) and Bf and Br (assemblage B) (Additional file 1: Table S1). These primers yielded 332-bp and 400-bp amplicons within the *tpi* locus of the assemblages A and B of *G. duodenalis*, respectively [52].

The *bg* locus of *G. duodenalis* was also amplified by nested PCR using external and internal forward and reverse oligonucleotide primers G7, G759, BG511F, and BG511R, respectively [54, 55]. The primary and secondary PCR reactions were performed in 50 µl reaction volumes as previously described [49]. The amplification program started with an initial denaturation step of 5 min at 95 °C, followed by 35 cycles of 30 s at 95 °C, 30 s at 65 °C, and 30 s at 72 °C with a final extension of 7 min at 72 °C [52]. In the second step of the PCR amplification, the annealing temperature was decreased to 55 °C, whereas other parameters were left unchanged.

The external forward and reverse oligonucleotide primers GDHeF and GDHiR, and internal forward GDHiF and reverse primer GDHiR were employed to amplify a 432-bp region of the *gdh* gene of *G. duodenalis* as indicated in Additional file 1: Table S1 [56]. The primary and secondary PCR reactions were performed in 50 µl reaction volumes as described [49].

The amplification scheme consisted of an initial step at 94 °C for 3 min, one cycle at 94 °C for 2 min, 61 °C for 1 min, and 68 °C for 2 min, followed by 30 amplification cycles at 94 °C for 30 s, 61 °C for 20 s, 68 °C for 20 s and a final extension at 68 °C for 7 min. The secondary PCR amplification consisted of an initial step at 94 °C for 3 min, one cycle at 94 °C for 2 min, 60 °C for 1 min, and 65 °C for 2 min, followed by 15 amplification cycles at 94 °C for 30 s, 60 °C for 20 s, 65 °C for 20 s with a final extension at 65 °C for 7 min.

The PCR products were size-fractionated on a 2% agarose gel in order to assess the amplification success and the DNA concentrations. The PCR-amplified products were further purified using calibrated AMPure^®^ XP magnetic beads, followed by the Illumina DNA library preparations. Purified PCR amplicons were subjected to sequencing analysis using a next-generation proprietary technology (bTEFAP^®^) on the Illumina MiSeq platform at Molecular Research LP (Shallowater, TX, USA), with the maximum expected error threshold set to 1.0.

### Phylogenetic analysis of the *tpi*, *gdh,* and *bg* loci of *G. duodenalis* clinical isolates

The amplicons yielded for each locus were directly subjected to sequencing analysis in both directions (Molecular Research LP, Shallowater, TX, USA). The DNA sequences were visualized and read by the CHROMAS program (Technelysium Pty Ltd., Queensland, Australia) and further aligned and assembled using DNASIS MAX (v. 3.0; Hitachi, Yokohama, Japan). The DNA sequences were blasted (http://blast.ncbi.nlm.nih.gov) against standard DNA sequences deposited in GenBank to compare sequence homology. The DNA sequences of the *tpi*, *bg*, and *gdh* loci were concatenated to obtain a single combined sequence for each *G. duodenalis* isolate successfully amplified at corresponding loci.

Phylogenetic trees were constructed using maximum likelihood with evolutionary distances calculated by the best-fitting model to describe a robust estimate of the evolutionary distances by MEGA X (www.megasoftware.net), and a subsequent 1000-replicate bootstrap to evaluate the reliability of clusters. The sequences obtained from this study have been deposited in GenBank under the accession numbers LC744999–LC745009 (*tpi*), LC745010–LC745020 (*bg*), and LC745021–LC745031 (*gdh*).

### Isolation and quantification of stool bacterial DNA

Total gDNA from fecal specimens was isolated using the QIAGEN DNeasy PowerSoil Pro Kit (Qiagen, Germantown, MD, USA), according to the manufacturer’s instructions. The quality and the quantity of isolated gDNA samples were determined with a Qubit™ 4 fluorometer using a Qubit™ dsDNA [double-stranded DNA] HS Assay Kit (Thermo Fisher Scientific, Waltham, MA, USA) according to the manufacturer’s specifications.

### Library preparation and the shotgun microbiome sequencing

DNA libraries were constructed using the Illumina Nextera XT DNA Library Preparation Kit (Illumina, San Diego, CA, USA), followed by indexing utilizing the Integrated DNA Technologies (IDT) unique dual indexes with a total gDNA input of 1 ng. The gDNA enzymatic fragmentation was performed using an Illumina Nextera XT kit (Illumina, San Diego, CA, USA). Subsequently, the unique dual indexes were added to each sample, followed by 12 cycles of PCR to construct libraries. The DNA libraries were further enriched using AMPure magnetic beads (Beckman Coulter) and eluted in Buffer EB (Qiagen). The purified DNA libraries were quantified using Qubit 4 fluorometer and Qubit^®^ dsDNA HS Assay Kit (Thermo Fisher Scientific) according to the manufacturer’s instructions. A paired-end sequencing (2 × 150-bp) was performed on the HiSeq X platform.

### Bioinformatics analysis

The HiSeq X platform utilizes a high-performance data-mining k-mer-based algorithm that efficiently groups millions of short sequence reads into distinct genome sequences. This platform consists of a pre-computation phase for reference databases and a per-sample computation phase. The inputs for the pre-computation phase are databases of reference genomes, virulence, and antimicrobial resistance markers, whereas the outputs for the pre-computational phase consist of phylogenetic relationships of microbes with a set of variable-length k-mer fingerprints. The per-sample computational phase, however, searches and compares hundreds of millions of short sequence reads or contigs from de novo assemblies against the fingerprint profiles. This query strategy enables the sensitive and accurate detection and the taxonomic classifications of microbial NGS reads, resulting in high-resolution taxonomic profiling and relative abundance estimates for the microbial NGS datasets. Relative abundance estimates were calculated by taking the abundance score for an individual taxon and dividing it by the total abundance score per group of samples. A filtering threshold based on the internal statistical scores derived from examining a large number of diverse metagenomes was further employed to exclude false positive identifications.

### Sequencing data analysis

Classified reads from CosmosID (CosmosID proprietary database; Germantown, MD, USA) were used to create a phyloseq object which was assessed for diversity metrics based on bacterial counts at the species level using the phyloseq (version 1.38.0) and microbial (https://cran.r-project.org/web/packages/microbial/index.html) R packages. Principal coordinate analysis (PCoA) plots using Bray–Curtis and Jaccard dissimilarity indices were generated based on genus-level bacterial read counts to identify differences between groups. Differential abundance analysis between groups was performed using the DESeq2 package (version 1.34.0) with a *P* < 0.05 and absolute log^2^fold change > 1 based on genus-level bacterial read counts.

### Pathway enrichment analysis

The raw paired-end sequencing data were obtained from CosmosID and assessed for quality using FastQC (version 0.11.9) and cleaned using Trimmomatic (version 0.39). Filtered reads were reassessed for quality using FastQC and classified taxonomically using Kraken2 (version 2.12) based on the Kraken2 PlusPF database (version 20210517). The Kraken2-generated, filtered FASTQ files were assigned to microbial metabolic pathways and functions using HUMAnN 3.04 (http://huttenhower.sph.harvard.edu/humann) and the UniRef90 diamond full reference database (version 201901b) [57]. Results from HUMAnN from each sample were evaluated for differences among the biological groups using the Kruskal–Wallis non-parametric test with a post hoc Dunn test.

### Fecal bile acid profile analysis

Bile acid (BA) internal standards, including taurocholic-d5, cholic-d4, glycocholic-d4 (1 ng/each), and 10 ng of chenodeoxycholic-d4 (Medical Isotopes, Inc., Pelham, NH, USA), were added to human fecal suspensions. Fecal suspensions were subsequently centrifuged at 12,000 g for 10 min and the soluble (fecal supernatants) and the insoluble (fecal pellets) fractions were dried under vacuum. The fecal pellets were used to calculate fecal dry weights for normalization. Pellets and dried supernatants were re-suspended in 50 µl of 75% methanol, and a total of 10 µl of the resuspension injected into the ultra-performance liquid chromatography–tandem mass spectrometry (UPLC-MS/MS) system for quantitative analysis.

The BAs were resolved on an ACQUITY UPLC HSS T3 column (1.8 µM, 100 Å pore diameter, 2.1 × 150 mm (Waters, Milford, MA, USA) with an ACQUITY UPLC HSS T3 precolumn (1.8 µM, 100 Å pore diameter, 2.1 × 5 mm) at 55 °C [58]. Solvents A (water) and B (acetonitrile) contained 0.1% formic acid. The BAs were eluted off the columns with an increasing gradient of solvent B from 39 to 98% at a flow rate of 0.45 ml/min as described earlier [59]. The BAs were analyzed using a triple quadrupole mass spectrometer (Xevo TQ-S, Waters) with electrospray ionization operated in negative ion mode. The MassLynx V4.1 software (Waters) was used for instrument control, acquisition, and sample analysis.

## Results

### Study population

Seventeen individuals, including eleven individuals infected with *G. duodenalis* (seven asymptomatic and four symptomatic individuals) and six healthy controls, were enrolled in this study. Infected individuals with asymptomatic human giardiasis did not show any overt clinical manifestations of human giardiasis, whereas those subjects with symptomatic giardiasis predominantly manifested gastrointestinal symptoms, including intermittent diarrhea, abdominal pain, and nausea, at the time of the diagnosis.

### The multilocus molecular characterization of *G. duodenalis* clinical isolates based on *tpi*, *bg*, and *gdh* loci: predominance of assemblage A (AII) of *G. duodenalis* in asymptomatic and symptomatic human subjects

The genetic heterogeneity of *G. duodenalis* clinical isolates from human subjects with asymptomatic and symptomatic giardiasis was determined using a multilocus analysis approach as described earlier [49–52]. The *G. duodenalis*-specific *tpi*, *bg*, and *gdh* loci were amplified using specific primers as described earlier [21, 51–55, 60–64]. None of these three loci were amplified in uninfected individuals initially identified as negative for *G. duodenalis* infection by microscopy on wet-mount preparations.

We found that a total of 8/11 (72.73%) human subjects were infected with assemblage A (sub-assemblage AII) of *G. duodenalis*, whereas 3/11 (27.27%) human subjects in the current study were infected with assemblage B of the parasite (Tables 2 and 3). Further analysis at the *tpi* and *gdh* loci demonstrated that all eight AII sub-assemblages belonged to the A2 (AII-A2) subtype. Analysis of the *bg* locus indicated that six clinical isolates belonged to the A3 subtype of *G. duodenalis* (AII-A3), whereas only two isolates were identified as the A2 subtype (AII-A2) (Table 2). Notably, all infected individuals harbored a single assemblage/genotype of *G. duodenalis* irrespective of their clinical profiles or whether they were symptomatic or asymptomatic, and no mixed infections at inter- (e.g., AI + AII, BII + BIV) or intra-assemblage levels (A + B) were detected (Tables 3 and 4).

**Table 2:** Identification of *G. duodenalis* assemblages, sub-assemblages, and subtypes based on analysis of *tpi*, *bg,* and *gdh* loci

Isolate	*tpi*	*bg*	*gdh*
ASYM_1	A (AII/A2)	A (AII/A3)	A (AII/A2)
ASYM_2	A (AII/A2)	A (AII/A2)	A (AII/A2)
ASYM_3	A (AII/A2)	A (AII/A3)	A (AII/A2)
ASYM_4	B	B	B
ASYM_5	B	B	B
ASYM_6	A (AII/A2)	A (AII/A3)	A (AII/A2)
ASYM_7	A (AII/A2)	A (AII/A3)	A (AII/A2)
SYM_1	A (AII/A2)	A (AII/A2)	A (AII/A2)
SYM_2	B	B	B
SYM_3	A (AII/A2)	A (AII/A3)	A (AII/A2)
SYM_4	A (AII/A2)	A (AII/A3)	A (AII/A2)

**Table 3:** *Giardia duodenalis* assemblage identification based on analysis of *tpi*, *bg,* and *gdh* loci

Genes	Assemblages *n* (%)		Total
	A	B	
*tpi*	8 (72.7)	3 (27.3)	11 (100.0)
*bg*	8 (72.7)	3 (27.3)	11 (100.0)
*gdh*	8 (72.7)	3 (27.3)	11 (100.0)

A: 8/11 (72.7%; 95% CI 43.4–90.3)

B: 3/11 (27.3%; 95% CI 9.7–56.6)

**Table 4:** Multilocus characterization of *G. duodenalis* parasites belonging to assemblage A based on the concatenated sequence alignments of the three sets of genes (*tpi*, *bg*, *gdh*)

MLG	Genotype			No. of isolates (isolate code)	GenBank accession no.		
	*tpi*	*bg*	*gdh*		*tpi*	*bg*	*gdh*
AII-1	A2	A2	A2	2 (ASYM_2; SYM_1)	U57897	AY072723	L40510, AY178737
AII-5	A2	A3	A2	4 (ASYM_1, 3, 7; SYM_4)	U57897	AY072724	L40510, AY178737

MLG multilocus genotype

Sequence alignments of the *tpi* loci of the eight clinical isolates belonging to assemblage A revealed two substitution patterns (Table 5). Seven isolates exhibited a 100% identity with AII reference sequences (U57897, KJ888993). However, a single isolate, SYM_3, showed an overlapping peak at nucleotide position 326 (G → R), which was not present in the sub-assemblage-defining positions, also known as the “hotspot sites” (Table 5). Sequence alignment utilizing the *tpi* locus also identified three isolates as belonging to assemblage, with three distinct nucleotide substitution patterns (Table 5). As presented in Table 5, the nucleotide sequences of all three isolates showed overlapping nucleotide peaks, making it impossible to make a sub-assemblage assignment. The presence of overlapping peaks could represent mixed infections or heterozygosity within the tetraploid genomes of *Giardia*. Assemblage B strains are reported to have higher levels of heterozygosity consistent with our observations.

**Table 5:** Multiple sequence alignments of the *tpi* locus from *G. duodenalis* isolates characterized in the current study in comparison with reference sequences retrieved from GenBank

	IsolatesGenBank accession no.	Nucleotide position from the start of the gene																										
Assemblage A		**129**	326	**399**																								
AI	KR051228	**T**	A	**C**																								
AI	L02120																											
AII-A2	U57897	**C**		**T**																								
AII-A2	KJ888993	**C**		**T**																								
AII-A2	ASYM_1, 2, 3, 6, 7; SYM_1, 4	C		T																								
AII-A2	SYM_3	C	R	T																								
Assemblage B		**39**	42	**91**	153	160	162	**165**	**168**	198	204	**210**	228	285	300	304	312	357	363	369	383	393	429	444	471	490	492	525
BIII	AF069561	**-**	-	**C**	T	G	G	**C**	**C**	C	T	**G**	C	A	T	A	C	C	T	T	C	C	G	C	A	A	C	C
BIII	AY368165	**G**	C													G						T						
BIV	AF069560	**A**	C	**T**				T	T			A											A					-
BIV	L02116	**A**	C	**T**				T	T			A																-
B	SYM_2	G	Y		Y		R			Y			Y	R	Y		S	M	K	K	Y			Y	R	R	S	Y
B	ASYM_5-*tpi* B	G	Y			Y					Y		Y		Y				K					Y			S	T
B	ASYM_4-*tpi* B	-	-	-	-	-	-	-	-	-	-	-	-	R	Y		G	A										T

Accession numbers of the isolates used as sub-assemblage reference isolates are in bold. Numbers in bold represent nucleotide substitutions from the start of the gene, which differentiate between sub-assemblages introduced by Weilinga and Thompson (2007) position and breakdown of intra-genotypic substitutions. Dots denote nucleotide homology with the AI (KR051228) or BIII (AY368165) reference sequence

Furthermore, the sequence analysis of the *bg* locus indicated a unique nucleotide substitution pattern, with one isolate exhibiting sequences homologous to subtype A3 with a single overlapping nucleotide peak at the 201 position (C → Y) (Table 6). This nucleotide substitution did not occur in the sub-assemblage-defining positions (i.e., "hotspot sites"), thereby further characterization at the sub-assemblage level was not attainable (Table 6). The multiple alignments based on the *bg* locus sequence analysis indicated three isolates being identified as assemblage B of *G. duodenalis*, each of which represented a unique nucleotide sequences pattern (Table 6). The nucleotide heterogeneity and subtype characterization of three isolates, with represented overlapping nucleotide peaks, are listed in Table 6.

**Table 6:** Multiple sequence alignments of the *bg* locus from *G. duodenalis* isolates characterized in the current study in comparison with reference sequences retrieved from GenBank

	Isolates/GenBank accession no.	Nucleotide position from the start of the gene																
Assemblage A		**174**	201	**349**	**460**	**468**	**483**	**541**	**606**									
AII-A2	AY072723	**G**	C	**T**	**C**	**T**	**T**	**G**	**T**									
AI-A1	X14185								**C**									
AI-A1	X85958								**C**									
AII-A3	AY072724				T	C												
AII-A4	AY545642							A	**C**									
AII-A5	AY545643						C		**C**									
AII-A6	AY545644			C					**C**									
AII-A7	AY545645	T																
AII-A8	AY545649						C		**C**									
AII**-**A2	ASYM_2; SYM_1																	
AII-A3	ASYM_1, 3, 7; SYM_3, 4				T	C												
AII-A3	ASYM_6		Y		T	C												
Assemblage B		177	184	**185**	**210**	**228**	**273**	**327**	**354**	**357**	418	**438**	**471**	495	**564**	**609**	**645**	**648**
BIII-B2	AY072726	T	A	**T**	**C**	**A**	**G**	**C**	**C**	**C**	A	**C**	**C**	C	**T**	**C**	**C**	**G**
BIV-B3	AY072727						A			T								
BIV-B1	AY072725				T		A	T	**T**	T		T						**A**
BIV-B4	AY072728					G	A		**T**	T					C	T	T	
BIV-B5	AY647265						A		**T**	T			T					
BIV-B6	AY647266			C	T		A			T								
B	ASYM_4		R				A			T	R			T			-	-
B	ASYM_5						A		Y	T							-	-
B	SYM_2	Y					A			T							-	-

Accession numbers of the isolates used as sub-assemblage reference isolates are included in bold. Numbers in bold represent nucleotide substitutions from the start of the gene, which differentiate between sub-assemblages introduced by Weilinga and Thompson (2007) and Cacciò et al. (2008) position and breakdown of intra-genotypic substitutions. Dots indicate nucleotide identity to the AII (AY072723) or BIII (AY072726) reference sequences

Eight isolates exhibited a 100% sequence homology with the A2 subtype (L40510) of *G. duodenalis* based on the multiple alignments of sequences obtained from the amplifications of the *gdh* locus (Table 7). Of those, 5 and 3 isolates were infecting individuals with asymptomatic and symptomatic giardiasis, respectively. Moreover, three isolates were identified as being assemblage B of *G. duodenalis*, with one and two isolates infected individuals with asymptomatic and symptomatic giardiasis, respectively (Table 7). Those isolates identified as being assemblage B of *G. duodenalis* represented three nucleotide substitution patterns as shown in Table 7.

**Table 7:** Multiple sequence alignments of the *gdh* locus from *G. duodenalis* isolates characterized in the current study in comparison with reference sequences retrieved from GenBank

	Isolates/GenBank accession no.	Nucleotide position from the start of the gene															
Assemblage A		**603**	**621**														
AI-A1	M84604	**T**	**C**														
AI-A1	KP687782																
AII-A2	L40510	**C**	**T**														
AII-A2	KJ741313	**C**	**T**														
AII-A2	ASYM_1, 2, 3, 6, 7; SYM_1, 3; 4	**C**	**T**														
Assemblage B		**249**	**297**	**309**	**357**	**360**	**429**	**447**	**519**	**540**	**561**	570	579	**597**	606	**612**	**666**
BIII	AF069059	**C**	**C**	**C**	**T**	**G**	**T**	**T**	**C**	**C**	**C**	C	C	**C**	C	**G**	**T**
BIII-like	DQ090541	**T**	**T**		**C**			**C**	**T**					**T**		**A**	**-**
BIV	L40508			**T**			**C**	**C**		**T**	**T**					**A**	
BIV	EU594666			**T**	**C**		**C**	**C**		**T**	**T**					**A**	**C**
BIV-like	AY826192			**T**	**C**		**C**	**C**		**T**							
B	ASYM_4	T		Y	C		Y	Y	Y	Y		Y		Y		R	C
B	ASYM_5	-			C		Y	C		T	Y				Y	R	-
B	SYM_2	-			C	A	C	C		T	Y	Y	M	Y		A	-

Accession numbers of the isolates used as sub-assemblage reference isolates are included in bold. Numbers in bold represent nucleotide substitutions from the start of the gene, which differentiate between sub-assemblages introduced by Weilinga and Thompson (2007) position and breakdown of intra-genotypic substitutions. Dots indicate nucleotide identity to the AII (L40510) or BIII (AF069059) reference sequences

Further sequence alignments using six concatenated sequences with unambiguous positions (i.e., no double peaks) indicated six isolates belonged to the assemblage A of *G. duodenalis*, representing two distinctive unique multilocus genotypes (MLGs; Table 4). Of these, two isolates were identified as being MLG AII-1, profile A2/A2/A2, whereas four isolates were demonstrated as being MLG AII-5, profile A2/A3/A2. Both of these two MLGs have been previously reported [17–19].

### *Giardia duodenalis* clinical isolates are closely related phylogenetically

**Fig. 1:**
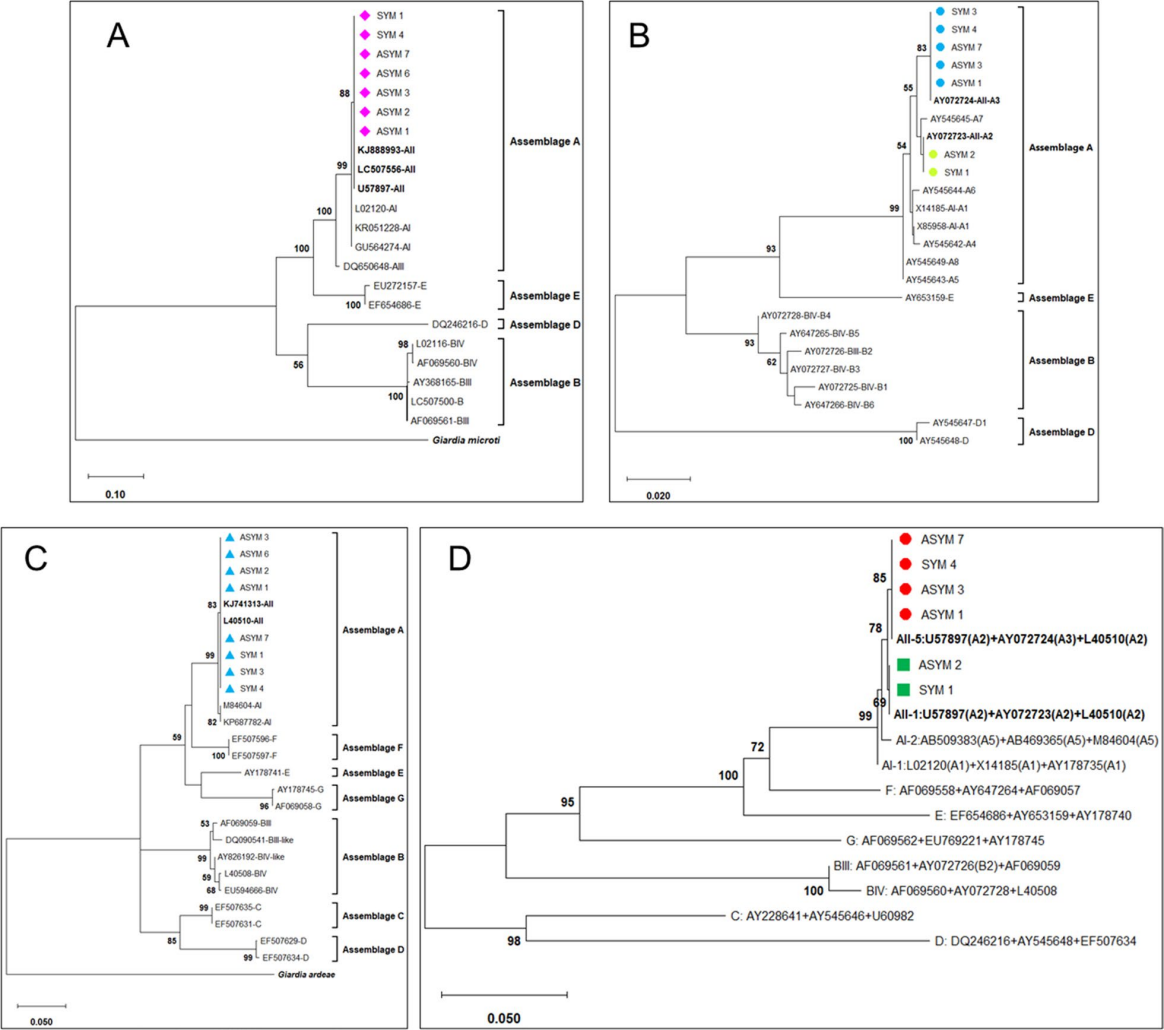
The phylogenetic relationships between *G. duodenalis* parasites from infected individuals with asymptomatic and symptomatic giardiasis. Clinical isolates from infected individuals with asymptomatic (*n* = 7) and symptomatic (*n* = 4) giardiasis were analyzed at the *tpi* (**A**), *bg* (**B**), and *gdh* (**C**) loci. The phylogenetic analysis based on the concatenated sequence alignments of the three sets of genes (*tpi*, *bg*, *gdh*) employed in an MLST-based characterization of *G. duodenalis* parasites (**D**). Magenta diamonds represent the A2 subtype (AII sub-assemblage) in 1A, and blue and green circles define the A3 subtype (AII sub-assemblage) and A2 subtype (AII sub-assemblages) in 1B, and blue triangles denote the A2 subtype (AII sub-assemblages) in 1C, respectively. Red circles represent AII-5 MLG, whereas green squares AII-2 MLG in 1D. In all phylogenetic trees, the percentage of trees achieved from 1000 replicates in which the associated taxa clustered together is shown next to the branches; only bootstrap values > 50% are demonstrated. The scale bar represents substitutions per nucleotide. Evolutionary analyses were conducted in MEGA X

Further phylogenetic analysis based on the *tpi* and *gdh* loci indicated that those clinical isolates belonging to assemblage A of *G. duodenalis* subjects clustered compactly together in a monophyletic clade despite being isolated from human subjects with asymptomatic and symptomatic human giardiasis (Fig. 1A and B). The construction of the phylogenetic tree based on the *bg* locus demonstrated that most *G. duodenalis* isolates from asymptomatic or symptomatic human subjects with giardiasis clustered together in a single monophyletic clade, whereas two isolates (one each from an asymptomatic and a symptomatic individual) were interspersed within two closely related adjacent clades (Fig. 1C). Consistently, the congruency phylogenetic analysis based on the three sets of concatenated genes (*tpi*, *bg*, *gdh*) further demonstrated the closely related genetic structures of *G. duodenalis* parasites irrespective of whether or not they were isolated from individuals with asymptomatic or symptomatic human giardiasis (Fig. 1D). Collectively, these findings suggested that *G. duodenalis* clinical isolates from both asymptomatic and symptomatic human subjects were genetically and evolutionary closely related and that the parasite genetic heterogeneity and complexity were unlikely the sole contributing factors defining the clinical outcome disparity observed during human giardiasis.

### Bacterial communities differ at the species level between *G. duodenalis*-infected individuals and healthy controls

**Fig. 2:**
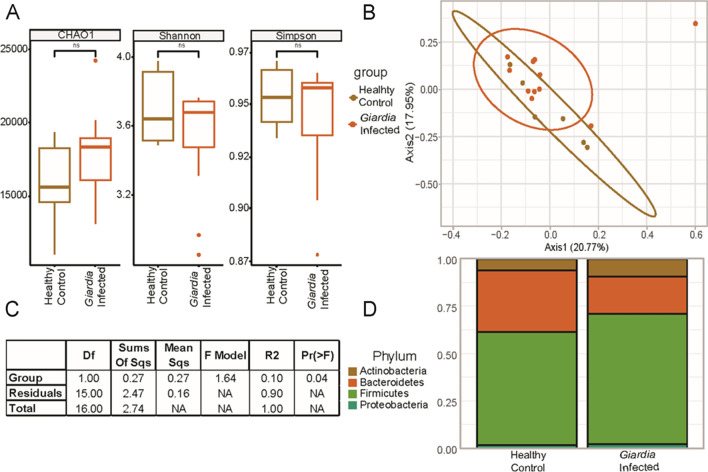
Microbial community diversity differences between healthy volunteers and infected patients with *G. duodenalis*. No significant differences were observed in intra-group microbiota diversity or richness (**A**). There is little separation in terms of beta diversity (Bray–Curtis Index) between healthy volunteers (*n* = 6) and infected (*n* = 11) individuals (**B**). From our permutational analysis of variance, a *P*-value of 0.04, suggests that there is a separation between healthy volunteers and *G. duodenalis*-infected patients (**C**). Relative abundance of the most prevalent phyla within groups also suggests shifts in the overall microbial composition (**D**)

Initially, we examined the differences between infected individuals and healthy controls in their microbiome diversity. The relative abundance of the most prevalent bacterial phyla was evaluated for diversity richness using alpha and beta diversity measures. The alpha diversity is focused on differences within groups, which was not significantly different between healthy controls and infected individuals in the current study (Fig. 2A). This indicates that the bacterial communities within each group had similar levels of diversity across samples. Furthermore, the beta diversity indices were used to evaluate the distance between *G. duodenalis*-infected individuals and healthy controls (Fig. 2B). While the distance did not reach our *P*-value cutoff for significance (*P* < 0.05), it did approach significance using the Bray–Curtis dissimilarity index (*P* = 0.051) (Fig. 2C). The Bray–Curtis dissimilarity index was used as a measure of dissimilarity between groups and subject to a permutational multivariate analysis of variance (PERMANOVA) test for significance. However, the permutational analysis of variance (*P* = 0.04) revealed a separation between healthy and *G. duodenalis*-infected patients (Fig. 2C).

While no significant differences were observed in intra-group microbiota diversity or richness between healthy controls and infected individuals, our analysis indicated shifts in the overall microbial composition between healthy controls and *G. duodenalis*-infected individuals. Our further analyses indicated an increase in the relative abundance of Actinobacteria and Proteobacteria, whereas the relative abundance of Bacteroidetes decreased following *G. duodenalis* infection as compared with healthy controls (Fig. 2D).

Our analysis of bacterial species enriched in healthy controls and *G. duodenalis*-infected individuals further indicated an association between the presence of *Enterococcus faecium* and the susceptibility to *G. duodenalis* infection in humans (Fig. 4A). In contrast, the presence of *Prevotella mizrahii* correlated inversely with susceptibility to *G. duodenalis*, as evidenced by the findings that this species was more abundant in healthy controls than those individuals with giardiasis (Fig. 4A).

### Shifts in microbiota communities at the species level related to the occurrence of symptomatic giardiasis

**Fig. 3:**
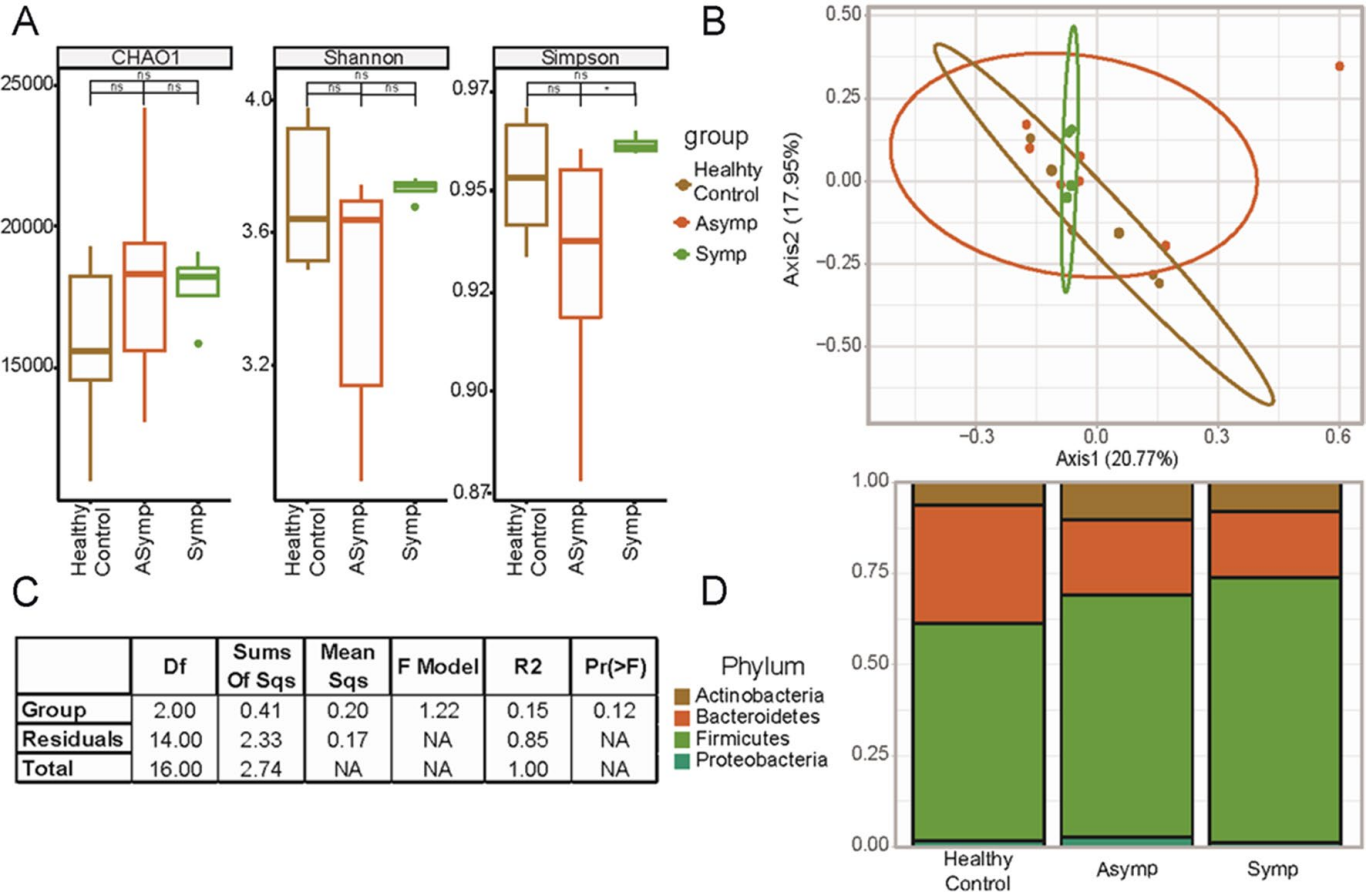
Microbial community diversity differences between healthy controls, asymptomatic, and symptomatic patients with giardiasis. Infected individuals were also examined in terms of the occurrence of symptoms and classified as asymptomatic (*n* = 7) and symptomatic (*n* = 4), then assessed for differences compared to healthy controls (*n* = 6). The only significant changes in intragroup microbiome diversity or richness were between asymptomatic and symptomatic patients (**A**). We also did not observe a significant change in beta diversity (Bray–Curtis Index) between our three groups (**B**). Permutational analysis of variance between groups reached a *P*-value of 0.12, which is above our threshold for significance (*P* < 0.05). **C**. The top phyla and their relative abundance within groups demonstrate some shifting proportions of the resident microbial population (**D**)

Further evaluation of differences within our data was also performed regarding three groups, including healthy controls, asymptomatic giardiasis infected, and symptomatic giardiasis infected. The microbiome diversity within groups was assessed by alpha diversity (richness, Simpson, and Shannon indices). We observed no significant differences in alpha diversity between groups (Fig. 3A). This indicates that within groups, the diversity of microbial communities is not changed. Complementary to this is the diversity of observed microbiota populations across groups which were evaluated by beta diversity (Bray and Jaccard dissimilarity index). No significant separations in beta diversity were identified across healthy control, asymptomatic, and symptomatic individuals (Fig. 3B and C).

Notably, we observed that the relative abundance of the phylum Proteobacteria increased following *G. duodenalis* infection as compared with healthy controls, with those with symptomatic giardiasis having the highest relative abundance of Proteobacteria, followed by individuals with asymptomatic giardiasis (Fig. 3D). Conversely, the relative abundance of Bacteroidetes declined with the development of symptomatic giardiasis, with those with symptomatic giardiasis having the lowest relative abundance of Bacteroidetes, followed by those individuals with asymptomatic giardiasis (Fig. 3D). The relative abundance of the other two phyla, Acinetobacter and Firmicutes, was not substantially altered between the groups, with Acinetobacter being more abundant in all groups as compared with Firmicutes.

**Fig. 4:**
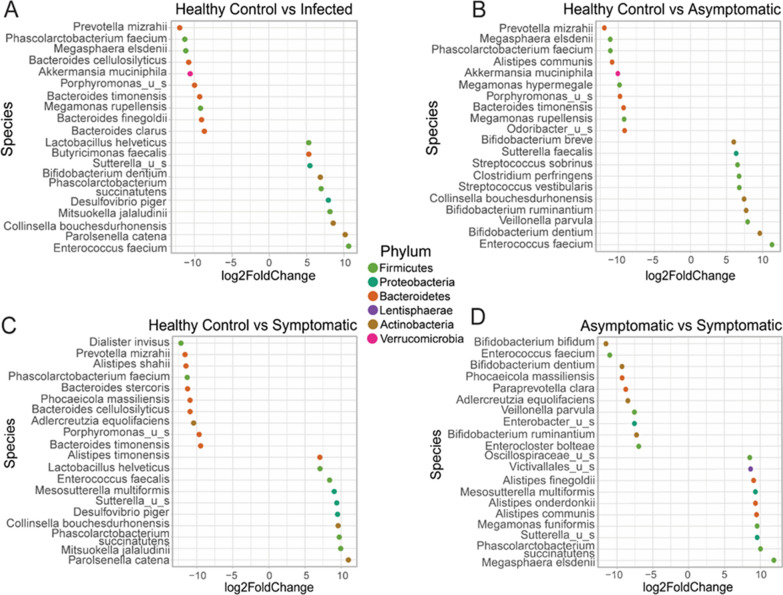
The top differential bacterial species within pairwise comparisons of groups. Pairwise investigation identified differentially abundant species between sample groups. The differential species in each dot plot were selected based on their log twofold change to include the ten most increased and ten most decreased species within each pairwise comparison. The comparisons performed include healthy volunteers and infected individuals (**A**), healthy volunteers and individuals with asymptomatic giardiasis (**B**), healthy volunteers and individuals with symptomatic giardiasis (**C**), as well as asymptomatic vs symptomatic (**D**). All species included in their relevant dot plots are statistically significant based on an adjusted *P* < 0.05

The pairwise comparisons between the groups revealed unique fecal bacterial community signatures between the groups (Fig. 4B–D). While *P. mizrahii* and *Megasphaera elsdenii* were more abundant in healthy volunteers, *E. faecium* was the most abundant bacterial species found in individuals with asymptomatic giardiasis, followed by *Bifidobacterium dentium* (Fig. 4B). When the bacterial signatures between healthy volunteers and individuals with the symptomatic disease were compared, we found that *Parolsenella catena*, followed *by Mitsuokella jalaludinii*, were enriched in individuals with symptomatic giardiasis (Fig. 4C). Notably, the bacterial community compositions were distinct between infected individuals with asymptomatic giardiasis as compared with those individuals with symptomatic disease (Fig. 4D).

**Fig. 5:**
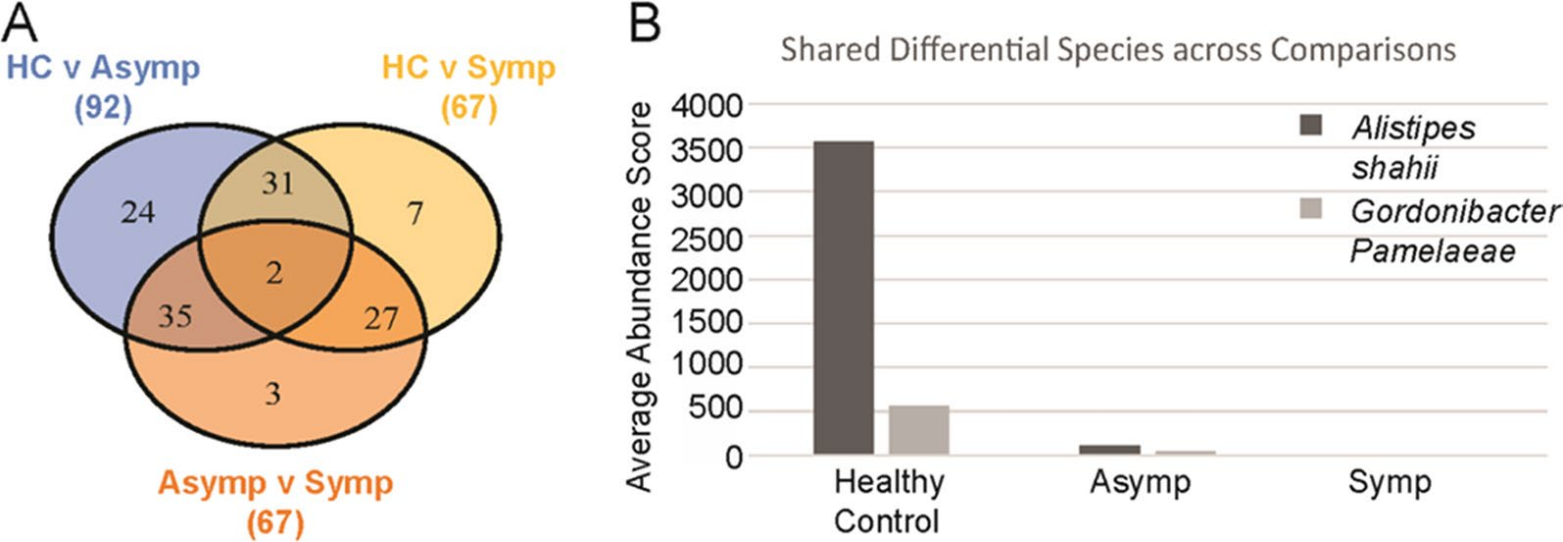
Overlap across significant differentially abundant bacterial species between pairwise comparisons. Significant differences between sample groups were determined using DESeq2 (adjusted *P* < 0.05). After determining the differential species between healthy controls (*n* = 6), and individuals with asymptomatic (*n* = 7), and symptomatic (*n* = 4) giardiasis, shared differentially abundance species across groups were determined. Numbers below each group title outside of the Venn diagram indicate the total number of significantly different bacterial species within each comparison (**A**). Shared differentially abundant species between groups and their average counts across samples are shown to demonstrate observed levels associated with patient status (**B**)

While our diversity measures, both within and between the healthy control, asymptomatic, and symptomatic fecal samples, were not significantly shifted within our study, we found that various bacteria demonstrated a statistically significant difference at the species level in our group pairwise comparisons. Comparing healthy controls and asymptomatic patient microbiota communities identified 92 total bacterial species as different between groups (Fig. 5A).

Similarly, when comparing samples from symptomatic patients and healthy controls, 67 total bacterial species were identified as meeting statistical significance between groups.

An interesting note is that although we can examine these samples as infected individuals and healthy controls rather than stratifying by the appearance of symptoms, we do not see a great amount of overlap between identified species. Only 31 shared bacterial species were significantly changed in both comparisons of asymptomatic and symptomatic groups to healthy controls. These bacterial communities separated by the appearance of symptoms, were also more evident when directly comparing asymptomatic and symptomatic samples, which had 67 differentially abundant bacterial species (Fig. 5).

Examining the overlap of identified species between pairwise group comparisons identified two which were significantly changed in all three comparisons. The shared bacterial species were *Alistipes shahii* and *Gordonibacter pamelaeae*, which are both decreased from healthy control to asymptomatic or symptomatic groups (Fig. 5B). These bacterial species could be of interest for their roles during giardiasis infection and their associations with the development of symptomatic disease.

### Assemblage-specific changes in the gut microbiome

**Fig. 6:**
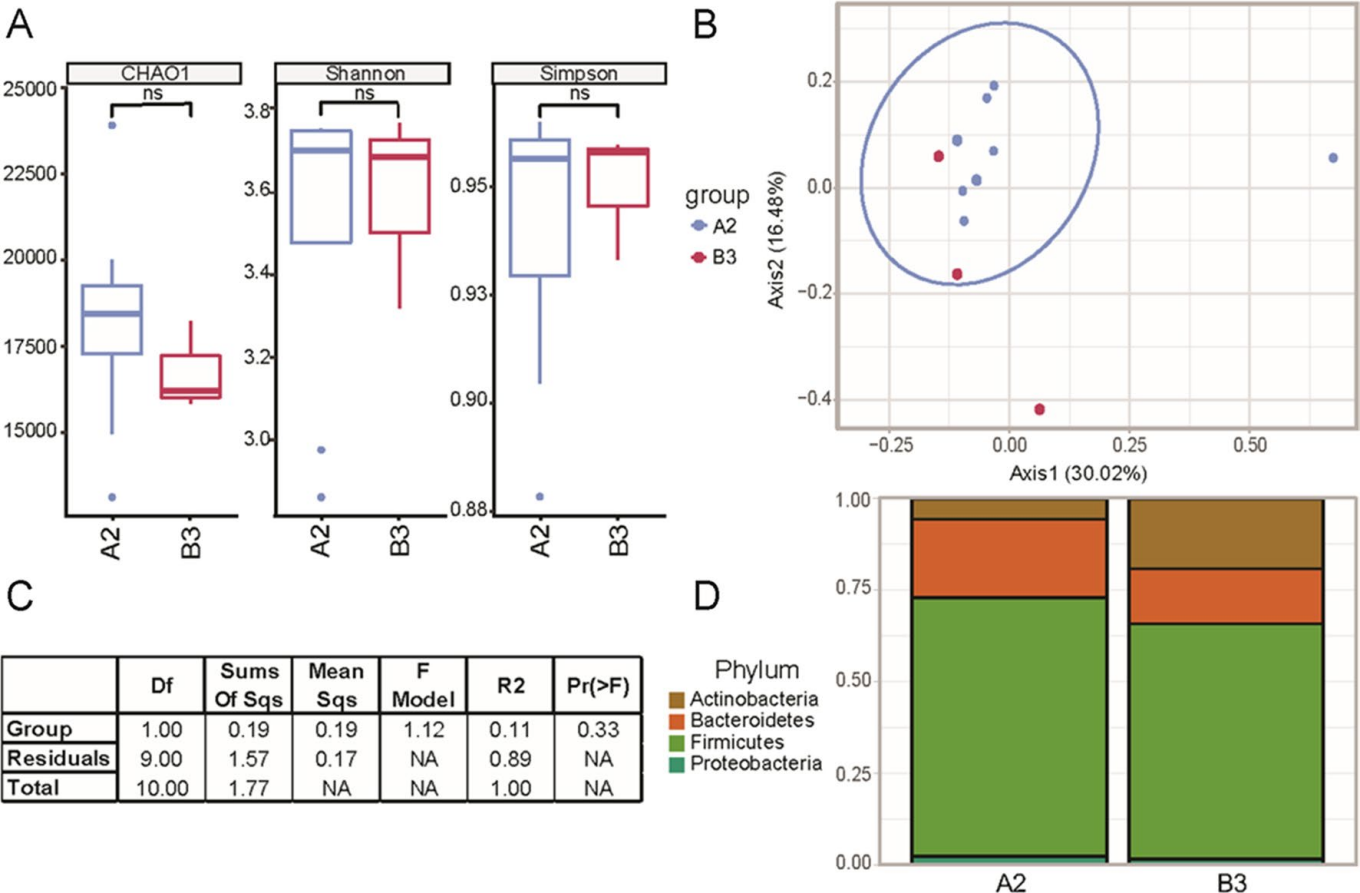
Microbial community diversity differences between *G. duodenalis* subtypes. Genotyping of *G. duodenalis* isolates revealed eight A2 and three B3 *G. duodenalis* across both asymptomatic and symptomatic patients. There we no significant changes in intragroup alpha diversity between the samples from patients with A2 or B3 *G. duodenalis* (**A**). There were also no significant changes in beta diversity (Bray–Curtis Index) between patients with A2 and B3 *G. duodenalis* (**B**). Permutation analysis of variance between groups reached a *P*-value of 0.33, which is well above our threshold for significance (*P* < 0.05) (**C**). The top phyla and their relative abundance within groups demonstrate some shifting proportions of the resident microbial population (D)

Insight into various assemblages of *G. duodenalis* and their relationship to resident microbiota were also examined between samples infected with assemblages A2 and B3 (Fig. 6). No significant differences were observed in alpha or beta diversity between these different subtypes. However, we observed a shift in phylum relative abundance between A2 and B3, as B3 had increased relative abundance in Actinobacteria and a decreased relative abundance of Bacteroidetes. However, this observation requires further investigation as the current data are limited by the number of samples, including eight A2 isolates and only three B3 isolates across both symptomatic and asymptomatic patients.

### Enriched pathways and functions represented by group microbial abundance profiles

Sequencing files were also subject to pathway enrichment analysis using HUMAnN 3.0 and the UniRef90 diamond full database (version 201901b). Overall, 95 pathways were significant across groups (*P* < 0.05). Many of these pathways are linked to metabolic degradation, biosynthesis, and recycling pathways. Pairwise comparisons of pathway results using Dunn’s test provided further insight into functional differences associated with giardiasis infection and symptomatic disease.

We found distinctive enriched metabolic pathway signatures associated with infected individuals (i.e., asymptomatic and symptomatic) as compared with healthy controls (Additional file 1: Tables S2–S5). As shown in Additional file 1: Table S2, we found that the 2-oxobutanoate degradation I pathway (*P* < 0.019) was differentially enriched between infected individuals (asymptomatic and symptomatic) as well as between individuals with asymptomatic giardiasis (*P* < 0.019) as compared with healthy controls. However, the pathway required for the biosynthesis of L-serine and glycine (L-serine and glycine biosynthesis I) was the main metabolic pathway enriched between individuals with symptomatic giardiasis and healthy controls (*P* < 0.03), followed by the 2-oxobutanoate degradation I pathway (*P* < 0.034). We also found that the C4 photosynthetic carbon assimilation cycle both nicotinamide adenine dinucleotide phosphate–malic enzyme (NADP-ME) type (*P* < 0.017) and phosphoenolpyruvate carboxykinase (PEPCK) type (*P* < 0.02) were significantly enriched between infected individuals with asymptomatic and symptomatic giardiasis (Additional file 1: Table S2).

### Fecal BA profiles are unaltered in individuals with giardiasis

We investigated the BA profiles in individuals infected with *G. duodenalis* in comparison with healthy controls using UPLC-MS/MS. Our analysis demonstrated detectable levels of primary (e.g., cholic acid, chenodeoxycholic acid) and secondary (e.g., deoxycholic acid, ursodeoxycholic acid, taurodeoxycholic acid) BAs in the feces of healthy volunteers (Additional file 2: Figure S1). Despite substantially higher concentrations of almost all primary and secondary BAs in the feces of *G. duodenalis*-infected individuals with asymptomatic giardiasis, the differences in the BAs concentrations between the three groups were not statistically significant.

## Discussion

In this study, we identified differences in the microbiome composition in human subjects with symptomatic and asymptomatic *G. duodenalis* infection as compared with healthy controls from the same population. We also found that the parasite’s genetic diversity was not associated with the clinical outcome of the infection. These findings were further confirmed by the observations that the majority of infected individuals were singly infected with genetically and evolutionary closely related *G. duodenalis* parasites. Pathway analysis indicated the enrichment of distinct metabolic pathways in the microbiome profiles of individuals with asymptomatic and symptomatic human giardiasis as compared with healthy controls, irrespective of the assemblages/genotypes of *G. duodenalis* found in these individuals. Collectively, our findings suggest the roles played by *G. duodenalis* assemblage- or genotype-independent parameters in determining the outcomes of clinical profiles in human giardiasis.

A long-lasting dilemma in the field of *Giardia* research is the mechanisms by which *G. duodenalis* causes clinical variability disease [2, 11, 12, 18]. Several hypotheses have been proposed as to why the overwhelming majority of human giardiasis cases are asymptomatic, whereas only a small portion of infected individuals develop clinical disease [11, 29–34]. Some studies have shown that a given *G. duodenalis* assemblage/genotype is associated with symptomatic disease, while others did not find such an association [14, 18–20]. In recent years, however, an increasing body of evidence has cast doubt on genetics being the sole contributing factor determining the clinical outcomes of human giardiasis [26, 61, 64].

*Giardia duodenalis* intimately interacts with the gut microbiome and the parasite/microbiome crosstalk determines host susceptibility to *G. duodenalis* infection, thereby defining the infection outcome [35, 40]. Notably, the microbiome undergoes alterations following infections with both assemblages A and B of *G. duodenalis* [41, 42], and these changes were dependent on the host's genetic background [42]. These shifts in the gut microbiome are observed throughout the entire intestinal tract of infected hosts and the resulting dysbiosis can persist even after the infection is resolved [43, 56, 65]. These observations suggest that the microbiome directly, through competing for colonization, or indirectly, via bacterial-derived metabolites, is capable of modulating host susceptibility and parasite virulence during giardiasis.

*Giardia* parasites employ a wide range of mechanisms by which they can subvert host immune responses in order to evade being effectively recognized following colonization in the small intestine [66–68]. The mucosal surface of the small intestine in human subjects infected with *G. duodenalis* lacks any overt signs of inflammation, even during the acute phase of infection [69]. This lack of pro-inflammatory immune responses could be further explained by the ability of *Giardia* parasites to modulate protective pro-inflammatory immune responses via competing for metabolites required for immune cell metabolism in the small intestine microenvironment [70]. For instance, the depletion of the amino acid arginine by *Giardia* parasites as a source of energy hinders the production of nitric oxide (NO) by intestinal epithelial cells (IECs), thereby impeding the proliferation of the IECs as well as compromising the ability of parasitized IECs to directly kill *Giardia* trophozoites [70, 71]. Despite the accumulation of macrophages and dendritic cells (DCs) in the lamina propria (LP) of the small intestine [72], *G. duodenalis* impairs the production of pro-inflammatory cytokines by macrophages (e.g., IL-8) and DCs in the LP of the small intestine following colonization [73, 74].

Our analyses demonstrated distinct microbial metabolic pathways between individuals infected with *G. duodenalis* (i.e., asymptomatic, symptomatic) as compared with healthy controls. It is well-established that multiple metabolites derived from bacterial metabolism, including secondary BAs, and short-chain fatty acids, have drastic immunomodulatory effects on the host and have been implicated in the pathogenesis of infectious and non-infectious diseases [75, 76].

We found that the 2-oxobutanoate degradation I pathway was differentially enriched between infected individuals (asymptomatic and symptomatic) as compared with healthy controls (Additional file 1: Table S2). The 2-oxobutanoate degradation I pathway is an intermediate pathway in the catabolism of multiple amino acids, including methionine and threonine [77, 78]. Methionine catalysis by the enzymes S-adenosylmethionine (SAM) synthetase or methionine adenosyltransferase (MAT) mainly occurs in the cytosol, leading to the generation of SAM [79, 80]. It is well-known that SAM is a pleiotropic molecule and functions as the main methyl group donor in methylation and regulates a wide range of biological processes, including transmethylation, trans-sulfuration, and polyamine synthesis [81].

Recent studies have demonstrated that SAM plays important roles in regulating immune homeostasis and T cell-dependent adaptive immunity by modulating the one-carbon metabolism in T cells [82]. SAM is a potent inhibitor of autophagy and promotes growth through the protein phosphatase 2A (PP2A) methylation, indicating the critical requirement of methionine and SAM levels in the regulation of autophagy [83, 84]. Based on these findings, it is likely that the methionine deprivation following *Giardia* infection limits SAM availability in the intestine leading to the promotion of autophagy and the lack of intestinal inflammation observed following *Giardia* infection. Furthermore, it is yet to be investigated whether *Giardia* infection hijacks the methionine metabolism and disrupts histone methylation in T cells, leading to T cell dysfunction during giardiasis, and whether the dietary supplementation of methionine or SAM alone or in combination will restore T cell dysfunction during giardiasis in humans or in murine models of human giardiasis.

## Study limitations

A few limitations, including limited sample size, restricted the interpretations of the findings of the current study. Moreover, this study was cross-sectional in design and, thereby did not reflect the dynamic nature of microbial changes over time during the course of *G. duodenalis* infection in humans. Future cohort studies with a larger sample size should clarify the roles played by the microbiome in varied clinical outcomes observed during human giardiasis.

## Conclusions

Our observations indicated the existence of distinct fecal microbial compositions as well as gut microbiome-derived bioactive metabolites between infected individuals with asymptomatic and symptomatic giardiasis in comparison with healthy controls. We also conclude that pathogen genetic heterogeneity is not likely the only contributing factor in determining the clinical outcomes of human giardiasis. Targeting the immunomodulatory microbiome-derived metabolites or their cognate binding receptors in the mammalian host’s intestinal tract may represent novel preventive or therapeutic targets for gut lumen-dwelling microbial pathogens.

## Supplementary Information

**Additional file 1: Figure S1**. The fecal bile acid profile in healthy volunteers as compared with infected individuals. The fecal profile of main primary and secondary bile acids in healthy controls (HC) and infected individuals with asymptomatic (Asym) and symptomatic (Sym) giardiasis were determined by the ultra-performance liquid chromatography-tandem mass spectrometry (UPLC-MS/MS) system for quantitative analysis.

**Additional file 2: Table S1**. Target genes and primer sequences utilized for identification of *G. duodenalis* assemblages and multilocus sequence analysis. **Table S2**. Pathways enriched between healthy controls vs. *G. duodenalis*-Infected individuals.**Table S3**. Pathways enriched between healthy controls vs. infected individuals with symptomatic giardiasis. **Table S4**. Pathways enriched between infected individuals with asymptomatic vs. symptomatic giardiasis. **Table S5**. Pathways enriched between healthy controls vs. infected individuals with asymptomatic giardiasis.

## Data Availability

The sequences obtained from this study have been deposited in GenBank under the accession numbers LC744999–LC745009 (*tpi*), LC745010–LC745020 (*bg*), and LC745021–LC745031 (*gdh*).

## Abbreviations

β2m: Beta-2 microglobulin
BA: Bile acid
*bg*: β-Giardin
DC: Dendritic cell
gDNA: Genomic DNA
*gdh*: Glutamate dehydrogenase
IEC: Intestinal epithelial cell
LP: Lamina propria
MLST: Multilocus sequence typing
NGS: Next-generation sequencing
NO: Nitric oxide
PCR: Polymerase chain reaction
PP2A: Protein phosphatase-2A
SAM: S-Adenosylmethionine
*tpi*: Triose phosphate isomerase
UPLC-MS/MS: Ultra-performance liquid chromatography–tandem mass spectrometry

## References

[1] Adam RD (2001). Biology of *Giardia lamblia*. Clin Microbiol Rev.

[2] Solaymani-Mohammadi S, Singer SM (2010). *Giardia duodenalis*: the double-edged sword of immune responses in giardiasis. Exp Parasitol.

[3] Solaymani-Mohammadi S (2022). Mucosal defense against *Giardia* at the intestinal epithelial cell interface. Front Immunol.

[4] Yoder JS, Gargano JW, Wallace RM, Beach MJ (2012). Giardiasis surveillance-United States, 2009–2010. MMWR Surveill Summ.

[5] Lane S, Lloyd D (2002). Current trends in research into the waterborne parasite *Giardia*. Crit Rev Microbiol.

[6] Thompson RC (2000). Giardiasis as a re-emerging infectious disease and its zoonotic potential. Int J Parasitol.

[7] Hellard ME, Sinclair MI, Hogg GG, Fairley CK (2000). Prevalence of enteric pathogens among community based asymptomatic individuals. J Gastroenterol Hepatol.

[8] Kappus KD, Lundgren RG, Juranek DD, Roberts JM, Spencer HC (1994). Intestinal parasitism in the United States: update on a continuing problem. Am J Trop Med Hyg.

[9] Scallan E, Hoekstra RM, Angulo FJ, Tauxe RV, Widdowson MA, Roy SL (2011). Foodborne illness acquired in the United States–major pathogens. Emerg Infect Dis.

[10] Coffey CM, Collier SA, Gleason ME, Yoder JS, Kirk MD, Richardson AM (2021). Evolving epidemiology of reported giardiasis cases in the United States, 1995–2016. Clin Infect Dis.

[11] Solaymani-Mohammadi S, Singer SM (2011). Host immunity and pathogen strain contribute to intestinal disaccharidase impairment following gut infection. J Immunol.

[12] Babaei Z, Malihi N, Zia-Ali N, Sharifi I, Mohammadi MA, Kagnoff MF (2016). Adaptive immune response in symptomatic and asymptomatic enteric protozoal infection: evidence for a determining role of parasite genetic heterogeneity in host immunity to human giardiasis. Microbes Infect.

[13] Nooshadokht M, Kalantari-Khandani B, Sharifi I, Kamyabi H, Liyanage NPM, Lagenaur LA (2017). Stool antigen immunodetection for diagnosis of *Giardia duodenalis* infection in human subjects with HIV and cancer. J Microbiol Methods.

[14] Cacciò SM, Lalle M, Svärd SG (2018). Host specificity in the *Giardia duodenalis* species complex. Infect Genet Evol.

[15] Feng Y, Xiao L (2011). Zoonotic potential and molecular epidemiology of *Giardia* species and giardiasis. Clin Microbiol Rev.

[16] Cai W, Ryan U, Xiao L, Feng Y (2021). Zoonotic giardiasis: an update. Parasitol Res.

[17] Woschke A, Faber M, Stark K, Holtfreter M, Mockenhaupt F, Richter J (2021). Suitability of current typing procedures to identify epidemiologically linked human *Giardia duodenalis* isolates. PLoS Negl Trop Dis.

[18] de Lucio A, Martínez-Ruiz R, Merino FJ, Bailo B, Aguilera M, Fuentes I (2015). Molecular genotyping of *Giardia duodenalis* isolates from symptomatic individuals attending two major public hospitals in Madrid. Spain PLoS One.

[19] Ahmad AA, El-Kady AM, Hassan TM (2020). Genotyping of *Giardia duodenalis* in children in upper Egypt using assemblage-specific PCR technique. PLoS ONE.

[20] Minetti C, Lamden K, Durband C, Cheesbrough J, Fox A, Wastling JM (2015). Determination of *Giardia duodenalis* assemblages and multi-locus genotypes in patients with sporadic giardiasis from England. Parasit Vectors.

[21] Lecová L, Weisz F, Tůmová P, Tolarová V, Nohýnková E (2018). The first multilocus genotype analysis of *Giardia intestinalis* in humans in the Czech Republic. Parasitology.

[22] Haque R, Roy S, Kabir M, Stroup SE, Mondal D, Houpt ER (2005). *Giardia* assemblage A infection and diarrhea in Bangladesh. J Infect Dis.

[23] Gelanew T, Lalle M, Hailu A, Pozio E, Cacciò SM (2007). Molecular characterization of human isolates of *Giardia duodenalis* from Ethiopia. Acta Trop.

[24] Puebla LJ, Núñez FA, Fernández YA, Fraga J, Rivero LR, Millán IA (2014). Correlation of *Giardia duodenalis* assemblages with clinical and epidemiological data in Cuban children. Infect Genet Evol.

[25] Pelayo L, Nuñez FA, Rojas L, Furuseth Hansen E, Gjerde B, Wilke H (2008). *Giardia* infections in Cuban children: the genotypes circulating in a rural population. Ann Trop Med Parasitol.

[26] Messa A, Köster PC, Garrine M, Gilchrist C, Bartelt LA, Nhampossa T, Massora S (2021). Molecular diversity of *Giardia duodenalis* in children under 5 years from the Manhiça district, Southern Mozambique enrolled in a matched case-control study on the aetiology of diarrhoea. PLoS Negl Trop Dis.

[27] Sarzhanov F, Köster PC, Dogruman-Al F, Bailo B, Dashti A, Demirel-Kaya F (2021). Detection of enteric parasites and molecular characterization of Giardia duodenalis and Blastocystis sp in patients admitted to hospital in Ankara Turkey. Parasitology.

[28] Köster PC, Malheiros AF, Shaw JJ, Balasegaram S, Prendergast A, Lucaccioni H (2021). Multilocus genotyping of *Giardia duodenalis* in mostly asymptomatic indigenous people from the Tapirapé Tribe. Brazilian Amazon Pathogens.

[29] Chin AC, Teoh DA, Scott KG, Meddings JB, Macnaughton WK, Buret AG (2002). Strain-dependent induction of enterocyte apoptosis by *Giardia lamblia* disrupts epithelial barrier function in a caspase-3-dependent manner. Infect Immun.

[30] Cevallos A, Carnaby S, James M, Farthing JG (1995). Small intestinal injury in a neonatal rat model of giardiasis is strain dependent. Gastroenterology.

[31] Solaymani-Mohammadi S, Singer SM (2013). Regulation of intestinal epithelial cell cytoskeletal remodeling by cellular immunity following gut infection. Mucosal Immunol.

[32] Scott KG, Logan MR, Klammer GM, Teoh DA, Buret AG (2000). Jejunal brush border microvillous alterations in *Giardia muris*-infected mice: role of T lymphocytes and interleukin-6. Infect Immun.

[33] Scott KG, Yu LC, Buret AG (2004). Role of CD8^+^ and CD4^+^ T lymphocytes in jejunal mucosal injury during murine giardiasis. Infect Immun.

[34] Keselman A, Li E, Maloney J, Singer SM (2016). The microbiota contributes to CD8^+^ T cell activation and nutrient malabsorption following intestinal infection with *Giardia duodenalis*. Infect Immun.

[35] Singer SM, Nash TE (2000). The role of normal flora in infections by *Giardia lamblia*. J Infect Dis.

[36] Torres MF, Uetanabaro APT, Costa AF, Alves CA, Farias LM, Bambirra EA (2000). Influence of bacteria from the duodenal microbiota of patients with symptomatic giardiasis on the pathogenicity of *Giardia duodenalis* in gnotoxenic mice. J Med Microbiol.

[37] Iebba V, Santangelo F, Totino V, Pantanella F, Monsia A, Di Cristanziano V (2016). Gut microbiota related to *Giardia duodenalis, Entamoeba* spp. and *Blastocystis hominis* infections in humans from Côte d'Ivoire. J Infect Dev Ctries.

[38] Halliez MC, Motta JP, Feener TD, Guérin G, LeGoff L, François A (2016). *Giardia duodenalis* induces paracellular bacterial translocation and causes postinfectious visceral hypersensitivity. Am J Physiol Gastrointest Liver Physiol.

[39] Mejia R, Damania A, Jeun R, Bryan PE, Vargas P, Juarez M (2020). Impact of intestinal parasites on microbiota and cobalamin gene sequences: a pilot study. Parasit Vectors.

[40] Berry ASF, Johnson K, Martins R, Sullivan MC, Farias Amorim C, Putre A (2020). Natural infection with *Giardia* is associated with altered community structure of the human and canine gut microbiome. mSphere.

[41] Pavanelli MF, Colli CM, Gomes ML, Góis MB, de Alcântara Nogueira de Melo G, de Almeida Araújo EJ, (2018). Comparative study of effects of assemblages AII and BIV of *Giardia duodenalis* on mucosa and microbiota of the small intestine in mice. Biomed Pharmacother.

[42] Yordanova IA, Cortés A, Klotz C, Kühl AA, Heimesaat MM, Cantacessi C (2019). RORγt^+^ Treg to Th17 ratios correlate with susceptibility to *Giardia* infection. Sci Rep.

[43] Chen TL, Chen S, Wu HW, Lee TC, Lu YZ, Wu LL (2013). Persistent gut barrier damage and commensal bacterial influx following eradication of *Giardia* infection in mice. Gut Pathog.

[44] Shukla G, Bhatia R, Sharma A (2016). Prebiotic inulin supplementation modulates the immune response and restores gut morphology in *Giardia duodenalis*-infected malnourished mice. Parasitol Res.

[45] Allain T, Chaouch S, Thomas M, Vallée I, Buret AG, Langella P (2018). Bile-salt-hydrolases from the probiotic strain *Lactobacillus johnsonii* La1 mediate anti-giardial activity in vitro and in vivo. Front Microbiol.

[46] Shukla G, Kamboj S, Sharma B (2020). Comparative analysis of antigiardial potential of heat inactivated and probiotic protein of probiotic *Lactobacillus rhamnosus* GG in murine giardiasis. Probiotics Antimicrob Proteins.

[47] Leitch GJ, Visvesvara GS, Wahlquist SP, Harmon CT (1989). Dietary fiber and giardiasis: dietary fiber reduces rate of intestinal infection by *Giardia lamblia* in the gerbil. Am J Trop Med Hyg.

[48] Allain T, Fekete E, Sosnowski O, Desmonts de Lamache D, Motta JP (2021). High-fat diet increases the severity of *Giardia* infection in association with low-grade inflammation and gut microbiota dysbiosis. Sci Rep.

[49] Hashemi-Hafshejani S, Meamar AR, Moradi M, Hemmati N, Solaymani-Mohammadi S, Razmjou E (2022). Multilocus sequence typing of *Giardia duodenalis* genotypes circulating in humans in a major metropolitan area. Front Med.

[50] Sulaiman IM, Fayer R, Bern C, Gilman RH, Trout JM, Schantz PM (2003). Triosephosphate isomerase gene characterization and potential zoonotic transmission of *Giardia duodenalis*. Emerg Infect Dis.

[51] Geurden T, Levecke B, Cacciò SM, Visser A, De Groote G, Casaert S (2009). Multilocus genotyping of *Cryptosporidium* and *Giardia* in non-outbreak related cases of diarrhoea in human patients in Belgium. Parasitology.

[52] Huey CS, Mahdy MAK, Al-Mekhlafi HM, Nasr NA, Lim YAL, Mahmud R (2013). Multilocus genotyping of *Giardia duodenalis* in Malaysia. Infect Genet Evol.

[53] Geurden T, Geldhof P, Levecke B, Martens C, Berkvens D, Casaert S (2008). Mixed *Giardia duodenalis* assemblage A and E infections in calves. Int J Parasitol.

[54] Cacciò SM, De Giacomo M, Pozio E (2002). Sequence analysis of the β-giardin gene and development of a polymerase chain reaction–restriction fragment length polymorphism assay to genotype *Giardia duodenalis* cysts from human faecal samples. Int J Parasitol.

[55] Lalle M, Pozio E, Capelli G, Bruschi F, Crotti D, Cacciò SM (2005). Genetic heterogeneity at the beta-giardin locus among human and animal isolates of *Giardia duodenalis* and identification of potentially zoonotic subgenotypes. Int J Parasitol.

[56] Barash NR, Maloney JG, Singer SM, Dawson SC (2017). *Giardia* alters commensal microbial diversity throughout the murine gut. Infect Immun.

[57] Beghini F, McIver LJ, Blanco-Míguez A, Dubois L, Asnicar F, Maharjan S (2021). Integrating taxonomic, functional, and strain-level profiling of diverse microbial communities with bioBakery. Elife.

[58] Kaur H, Seeger D, Golovko S, Golovko M, Combs CK. Liver Bile acid changes in mouse models of Alzheimer's Disease. Int J Mol Sci. 2021;22(14):7451. 10.3390/ijms22147451 PMC8303891 34299071

[59] Kaur H, Nagamoto-Combs K, Golovko S, Golovko MY, Klug MG, Combs CK (2020). Probiotics ameliorate intestinal pathophysiology in a mouse model of Alzheimer’s disease. Neurobiol Aging.

[60] Read CM, Monis PT, Thompson RC (2004). Discrimination of all genotypes of *Giardia duodenalis* at the glutamate dehydrogenase locus using PCR-RFLP. Infect Genet Evol.

[61] Sahagún J, Clavel A, Goñi P, Seral C, Llorente MT, Castillo FJ (2008). Correlation between the presence of symptoms and the *Giardia duodenalis* genotype. Eur J Clin Microbiol Infect Dis.

[62] Elhadad H, Abdo S, Tolba M, Salem AI, Mohamed MA, El-Abd EA (2021). Detection of *Giardia intestinalis* assemblages A and B among children from three villages in the West Delta region, Egypt using assemblage specific primers. J Parasit Dis.

[63] Fahmy HM, El-Serougi AO, El Deeb HK, Hussein HM, Abou-Seri HM, Klotz C (2015). *Giardia duodenalis* assemblages in Egyptian children with diarrhea. Eur J Clin Microbiol Infect Dis.

[64] Tamer GS, Kasap M, Er DK (2015). Genotyping and phylogenetic analysis of *Giardia duodenalis* isolates from Turkish children. Med Sci Monit.

[65] Beatty JK, Akierman SV, Motta JP, Muise S, Workentine ML, Harrison JJ (2017). *Giardia duodenalis* induces pathogenic dysbiosis of human intestinal microbiota biofilms. Int J Parasitol.

[66] Kamda JD, Singer SM (2009). Phosphoinositide 3-kinase-dependent inhibition of dendritic cell interleukin-12 production by *Giardia lamblia*. Infect Immun.

[67] Banik S, Renner Viveros P, Seeber F, Klotz C, Ignatius R, Aebischer T (2013). *Giardia duodenalis* arginine deiminase modulates the phenotype and cytokine secretion of human dendritic cells by depletion of arginine and formation of ammonia. Infect Immun.

[68] Rodríguez-Walker M, Molina CR, Luján LA, Saura A, Jerlström-Hultqvist J, Svärd SG (2022). Comprehensive characterization of cysteine-rich protein-coding genes of *Giardia lamblia* and their role during antigenic variation. Genomics.

[69] Oberhuber G, Kastner N, Stolte M (1997). Giardiasis: a histologic analysis of 567 cases. Scand J Gastroenterol.

[70] Schofield PJ, Costello M, Edwards MR, O'Sullivan WJ (1990). The arginine dihydrolase pathway is present in *Giardia intestinalis*. Int J Parasitol.

[71] Eckmann L, Laurent F, Langford TD, Hetsko ML, Smith JR, Kagnoff MF (2000). Nitric oxide production by human intestinal epithelial cells and competition for arginine as potential determinants of host defense against the lumen-dwelling pathogen *Giardia lamblia*. J Immunol.

[72] Li E, Zhou P, Singer SM (2006). Neuronal nitric oxide synthase is necessary for elimination of *Giardia lamblia* infections in mice. J Immunol.

[73] Maloney J, Keselman A, Li E, Singer SM (2015). Macrophages expressing arginase 1 and nitric oxide synthase 2 accumulate in the small intestine during *Giardia lamblia* infection. Microbes Infect.

[74] Liu J, Ma'ayeh S, Peirasmaki D, Lundström-Stadelmann B, Hellman L, Svärd SG (2018). Secreted *Giardia intestinalis* cysteine proteases disrupt intestinal epithelial cell junctional complexes and degrade chemokines. Virulence.

[75] Ma'ayeh SY, Liu J, Peirasmaki D, Hörnaeus K, Bergström Lind S, Grabherr M (2017). Characterization of the *Giardia intestinalis* secretome during interaction with human intestinal epithelial cells: the impact on host cells. PLoS Negl Trop Dis.

[76] Campbell C, McKenney PT, Konstantinovsky D, Isaeva OI, Schizas M, Verter J (2020). Bacterial metabolism of bile acids promotes generation of peripheral regulatory T cells. Nature.

[77] Hang S, Paik D, Yao L, Kim E, Trinath J, Lu J (2019). Bile acid metabolites control T_H_17 and T_reg_ cell differentiation. Nature.

[78] Rébeillé F, Jabrin S, Bligny R, Loizeau K, Gambonnet B, Van Wilder V, Douce R (2006). Methionine catabolism in *Arabidopsis* cells is initiated by a gamma-cleavage process and leads to S-methylcysteine and isoleucine syntheses. Proc Natl Acad Sci USA.

[79] Pan Y, Hu F, Yu C, Li C, Huang T, Hu H (2020). Amino acid catabolism during nitrogen limitation in *Phaeodactylum tricornutum*. Front Plant Sci.

[80] Kocsis MG, Ranocha P, Gage DA, Simon ES, Rhodes D, Peel GJ (2003). Insertional inactivation of the methionine s-methyltransferase gene eliminates the s-methylmethionine cycle and increases the methylation ratio. Plant Physiol.

[81] Schalk-Hihi C, Markham GD (1999). The conformations of a substrate and a product bound to the active site of S-adenosylmethionine synthetase. Biochemistry.

[82] Lu SC, Mato JM (2008). S-adenosylmethionine in cell growth, apoptosis and liver cancer. J Gastroenterol Hepatol.

[83] Yerinde C, Siegmund B, Glauben R, Weidinger C (2019). Metabolic control of epigenetics and its role in CD8^+^ T cell differentiation and function. Front Immunol.

[84] Sutter BM, Wu X, Laxman S, Tu BP (2013). Methionine inhibits autophagy and promotes growth by inducing the SAM-responsive methylation of PP2A. Cell.

