# Supplementary material for: A shotgun metagenomic analysis of the fecal microbiome in humans infected with Giardia duodenalis
Source: Parasit Vectors. 2023 Jul 18;16:239. doi: 10.1186/s13071-023-05821-1 (PMC10354925; doi:10.1186/s13071-023-05821-1)
Supplement: Supplementary file 1 — Additional file 1: Figure S1. The fecal bile acid profile in healthy volunteers as compared with infected individuals. The fecal profile of main primary and secondary bile acids in healthy controls (HC) and infected individuals with asymptomatic (Asym) and symptomatic (Sym) giardiasis were determined by the ultra-performance liquid chromatography-tandem mass spectrometry (UPLC-MS/MS) system for quantitative analysis. [file 13071_2023_5821_MOESM1_ESM.docx]

**Table S1.** Target genes and primer sequences utilized for identification of *G. duodenalis* assemblages and multilocus sequence analysis.

| **Target gene** | **Nested PCR Primer designation and nucleotide Sequences (5’–3’)** | | **Amplicon size (bp)** | **Reference** |
| --- | --- | --- | --- | --- |
|  | **External primers** | **Internal primers** |  |  |
| ***tpi*** | AL3543: AAATIATGCCTGCTCGTCG | AL3544: CCCTTCATCGGIGGTAACTT | 530 | [57] |
|  | AL3546: CAAACCTTITCCGCAAACC | AL3545: GTGGCCACCACICCCGTGCC |  |  |
| ***tpi*** |  | Af: CGCCGTACACCTGTCA | 332 | [60,61] |
|  | AL3543: AAATIATGCCTGCTCGTCG | Ar: AGCAATGACAACCTCCTTCC |  |  |
|  | AL3546: CAAACCTTITCCGCAAACC | Bf: GTTGTTGTTGCTCCCTCCTTT | 400 |  |
|  |  | Br: CCGGCTCATAGGCAATTACA |  |  |
| ***bg*** | G7: AAGCCCGACGACCTCACCCGCAGTGC | BG511F: GAACGAACGAGATCGAGGTCCG | 511 | [62,63] |
|  | G759: GAGGCCGCCCTGGATCTTCGAGACGAC | BG511R: CTCGACGAGCTTCGTGTT |  |  |
| ***gdh*** | GDHeF: TCAACGTYAAYCGYGGYTTCCGT | GDHiF: CAGTACAACTCYGCTCTCGG | 432 | [64] |
|  | GDHiR: GTTRTCCTTGCACATCTCC | GDHiR: GTTRTCCTTGCACATCTCC |  |  |

**Table S2**. Pathways enriched between healthy controls vs. *G. duodenalis*-Infected individuals

| Pathway | Chi^2^ | HC vs. Sym | |
| --- | --- | --- | --- |
|  |  | *p* | *p*adj |
| PWY-8073: lipid IVA biosynthesis (*Pseudomonas putida*) | 4.455 | 0.035 | 0.953 |
| NAGLIPASYN-PWY: lipid IVA biosynthesis (*Escherichia coli*) | 4.455 | 0.035 | 0.953 |
| PWY-5130: 2-oxobutanoate degradation I | 4.455 | 0.035 | 0.953 |

**Table S3**. Pathways enriched between healthy controls vs. infected individuals with symptomatic giardiasis

| Pathway | Chi^2^ | HC vs. Sym | | |
| --- | --- | --- | --- | --- |
|  |  | Z | *p* | *p*adj |
| SER-GLYSYN-PWY: super pathway of L-serine and glycine biosynthesis I | 5.451 | 2.326 | 0.010 | 0.030 |
| PWY-5130: 2-oxobutanoate degradation I | 6.991 | 2.284 | 0.011 | 0.034 |
| PWY-5989: stearate biosynthesis II (bacteria and plants) | 5.241 | 2.281 | 0.011 | 0.034 |
| PENTOSE-P-PWY: pentose phosphate pathway | 5.198 | 1.981 | 0.024 | 0.036 |
| PWY-6859: all-trans-farnesol biosynthesis | 6.216 | 1.960 | 0.025 | 0.037 |
| PWY-7312: dTDP-&beta;-D-fucofuranose biosynthesis | 6.216 | 1.960 | 0.025 | 0.037 |
| PWY-7392: taxadiene biosynthesis (engineered) | 6.216 | 1.960 | 0.025 | 0.037 |
| PWY-5659: GDP-mannose biosynthesis | 5.718 | 2.224 | 0.013 | 0.039 |
| PWY-7851: coenzyme A biosynthesis II (eukaryotic) | 4.957 | 2.224 | 0.013 | 0.039 |
| PWY-7323: super pathway of GDP-mannose-derived O-antigen building blocks biosynthesis | 4.650 | 2.148 | 0.016 | 0.048 |

**Table S4**. Pathways enriched between infected individuals with asymptomatic vs. symptomatic giardiasis.

| Pathway | Chi^2^ | Asym vs. Sym | | |
| --- | --- | --- | --- | --- |
|  |  | Z | *p* | *p*adj |
| PWY-241: C4 photosynthetic carbon assimilation cycle, NADP-ME type | 6.517 | 2.526 | 0.006 | 0.017 |
| PWY-7117: C4 photosynthetic carbon assimilation cycle, PEPCK type | 6.211 | 2.478 | 0.007 | 0.020 |
| PWY-5384: sucrose degradation IV (sucrose phosphorylase) | 5.562 | 2.358 | 0.009 | 0.028 |
| PWY-5659: GDP-mannose biosynthesis | 5.718 | 2.065 | 0.019 | 0.029 |
| PWY-7111: pyruvate fermentation to isobutanol (engineered) | 5.361 | 2.313 | 0.010 | 0.031 |
| PWY-5103: L-isoleucine biosynthesis III | 5.032 | 2.223 | 0.013 | 0.039 |
| NAD-BIOSYNTHESIS-II: NAD salvage pathway III (to nicotinamide riboside) | 4.950 | 2.163 | 0.015 | 0.046 |
| PWY-6151: S-adenosyl-L-methionine salvage I | 4.655 | 2.155 | 0.016 | 0.047 |
| PWY-6936: seleno-amino acid biosynthesis (plants) | 4.637 | 2.144 | 0.016 | 0.048 |

**Table S5**. Pathways enriched between healthy controls vs. infected individuals with asymptomatic giardiasis.

| Pathway | Chi^2^ | HC vs. Asym | | |
| --- | --- | --- | --- | --- |
|  |  | Z | *p* | *p*adj |
| PWY-5130: 2-oxobutanoate degradation I | 6.991 | -2.240 | 0.013 | 0.019 |
| PWY-6859: all-trans-farnesol biosynthesis | 6.216 | -2.274 | 0.011 | 0.034 |
| PWY-7312: dTDP-&beta;-D-fucofuranose biosynthesis | 6.216 | -2.274 | 0.011 | 0.034 |
| PWY-7392: taxadiene biosynthesis (engineered) | 6.216 | -2.274 | 0.011 | 0.034 |
| NAGLIPASYN-PWY: lipid IVA biosynthesis (*E. coli*) | 5.158 | -2.268 | 0.012 | 0.035 |
| PWY-8073: lipid IVA biosynthesis (*P. putida*) | 5.158 | -2.268 | 0.012 | 0.035 |
