# Supplementary figures and images for: A shotgun metagenomic analysis of the fecal microbiome in humans infected with Giardia duodenalis
Source: Parasit Vectors. 2023 Jul 18;16:239. doi: 10.1186/s13071-023-05821-1 (PMC10354925; doi:10.1186/s13071-023-05821-1)

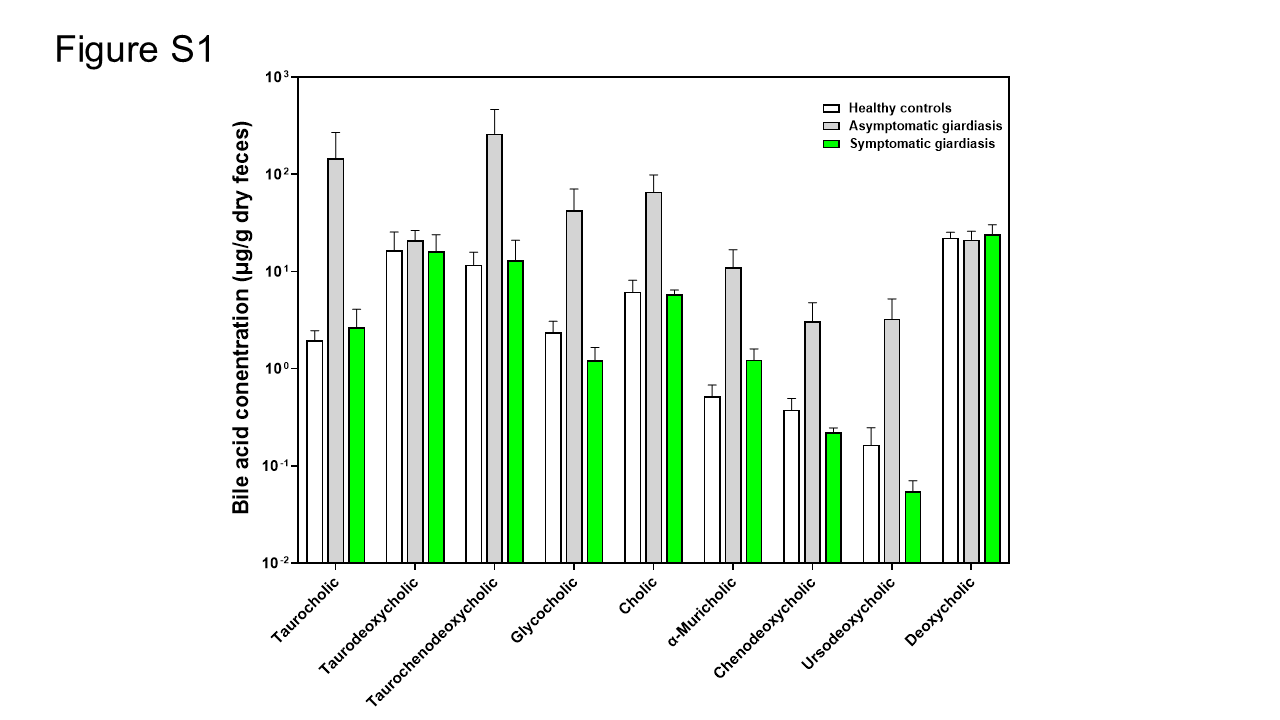

Supplement: Supplementary file 2 — Additional file 2: Table S1. Target genes and primer sequences utilized for identification of G. duodenalis assemblages and multilocus sequence analysis. Table S2. Pathways enriched between healthy controls vs. G. duodenalis-Infected individuals.Table S3. Pathways enriched between healthy controls vs. infected individuals with symptomatic giardiasis. Table S4. Pathways enriched between infected individuals with asymptomatic vs. symptomatic giardiasis. Table S5. Pathways enriched between healthy controls vs. infected individuals with asymptomatic giardiasis. [file 13071_2023_5821_MOESM2_ESM.tif]
